# The Effectiveness of Manual Therapy in the Cervical Spine and Diaphragm, in Combination with Breathing Reeducation Exercises, in Patients with Non-Specific Chronic Neck Pain: Protocol for Development of Outcome Measures and a Randomized Controlled Trial

**DOI:** 10.3390/diagnostics12112690

**Published:** 2022-11-04

**Authors:** Petros I. Tatsios, Eirini Grammatopoulou, Zacharias Dimitriadis, George A. Koumantakis

**Affiliations:** 1 Physiotherapy Department, School of Health & Care Sciences, University of West Attica (UNIWA), 12243 Athens, Greece; 2 Laboratory of Advanced Physiotherapy, Physiotherapy Department, School of Health & Care Sciences, University of West Attica (UNIWA), 12243 Athens, Greece; 3 Health Assessment & Quality of Life Laboratory, Physiotherapy Department, University of Thessaly, 35100 Lamia, Greece

**Keywords:** chronic neck pain, manual therapy, diaphragm, breathing exercises, respiratory dysfunction, breathing reeducation

## Abstract

Until now, non-specific chronic neck pain has mainly been considered as a musculoskeletal system dysfunction, with associated psychological involvement due to its prolonged or recurrent nature. However, patients with non-specific chronic neck pain frequently additionally exhibit respiratory dysfunction. Emerging evidence suggests that addressing the respiratory dysfunction in these patients will provide additional therapeutic benefits in musculoskeletal and respiratory-related outcomes for several reasons (biomechanical, biochemical, and psychological). Motor control dysfunction of the muscles surrounding the spine (diaphragm included) negatively affects the mechanics and biochemistry of breathing (pH-homeostasis). An impaired and ineffective breathing pattern has been recognized as the primary source of many unexplained symptoms (anxiety, depression, confusion, chest pain, hypocapnia, and breathlessness) in patients with non-specific chronic neck pain. The proposed protocol’s purpose is dual: to assess the relative effectiveness of manual therapy in the cervical spine and the diaphragm, in combination with breathing reeducation exercises, along with cervical spine manual therapy or usual physical therapy care on the underlying dysfunctions in patients with non-specific chronic neck pain via a randomized controlled clinical trial, and to validate part of the outcome measures. Several musculoskeletal and respiratory dysfunction outcomes will be employed to delimit the initial extent and level of dysfunction and its resolution with the treatments under study.

## 1. Introduction

Chronic neck pain is one of the most commonly reported musculoskeletal pathologies in the general population [1]. It has an immense impact on the physical, social, and psychological aspects and quality of life of the individual and society as a whole [1]. A total of 223 million people are affected worldwide, and 22 million live for years with neck pain-related disability, according to the Global Burden of Diseases, Injuries, and Risk Factors Study 2019 [2]. The primary pathology and pathophysiology of chronic neck pain are still unclear and significantly contribute to morbidity and disability in everyday life and at work in many countries [1,3]. 

Mechanical neck pain is defined as pain and discomfort localized between the superior nuchal line, cervical spine, and the spinous process of the first thoracic vertebra. The pain may refer to another region, such as the scapula, anterior chest wall, head, or upper limb [4]. Chronic neck pain describes neck pain persisting for longer than 12 weeks or after the healing period, or recurring neck pain that intermittently affects an individual over a long period of time [5]. The anatomical sources of the pain appear to be several (facet joints, intervertebral discs, and myofascial tissues). Since, on most occasions, clinicians cannot identify the exact source of the pain, the pain is characterized as non-specific, and the diagnosis is based mainly on patients’ clinical signs and symptoms [6]. Clinicians must differentiate chronic neck pain related to serious pathologies (e.g., rheumatoid arthritis, metastatic bone disease) from biomechanical-related neck disorders [6]. 

**Figure 1:**
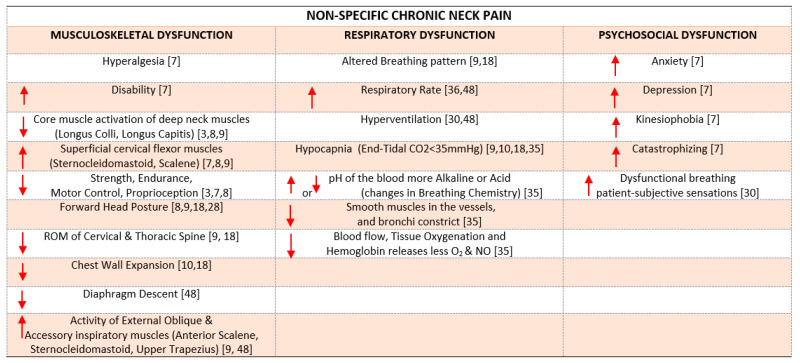
The three aspects of chronic neck pain dysfunction, with corresponding references. (red arrow mean increase or decrease).

People with non-specific chronic neck pain (NSCNP) present with local hyperalgesia, impaired conditioned pain modulation, low quality of life, and psychological disruptions (fear of movement, depressive symptoms, pain catastrophizing) [7]. Furthermore, cervical muscles not only stabilize the cervical spine, but contribute to respiration (sternocleidomastoid, anterior scalene, trapezius). These muscles frequently display diminished strength, endurance, motor control, and proprioception as a result of pain [7,8]. Neck pain may result in altered neck muscle activation levels and variable neck muscle activation sequences (incoordination patterns), including reduced participation of the deep segmental neck muscles and increased participation of the superficial neck muscles [3,8]. The above-mentioned pain-related dysfunctions contribute to forward head posture (FHP) maintenance, reduced active range of motion, and a poor and altered breathing patterns [9]. In consequence, the chemistry (pH blood levels) and respiration pattern may change, causing smooth respiratory muscle constriction, altered electrolyte balance, decreased tissue oxygenation [10], increased excitability in the nervous and muscular system, hypertonia of the accessory muscles, hypomobility of the thoracic cage, shortening of the accessory muscles, and a vicious circle contributing to the pain experienced in NSCNP [9,10]. Dysfunctional breathing (DB) presents in the form of changes in the blood chemistry, dysfunction of respiratory muscles, and a decrease in respiratory muscle strength and associated respiratory outcomes, such as maximum expiratory pressure (MEP), maximum inspiratory pressure (MIP), end tidal CO_2_ (ETCO_2_), forced expiratory volume in the 1st second of expiration (FEV1), maximum voluntary ventilation (MVV), and forced vital capacity (FVC) [8,9,10]. The three aspects of dysfunction contributing to NSCNP are presented in Figure 1.

Due to the complexity of chronic neck pain, mainly due to the diversity of factors associated with the development or persistence of neck pain, the treatment resources used in the rehabilitation of NSCNP vary, and there is conflicting evidence about the most appropriate therapeutic modalities [6]. Although patients with NSCNP have conventionally been treated by traditional physical therapy, manual therapy (spinal mobilization, manipulation techniques), education, or exercise [11], some do not experience complete recovery, suggesting the need for additional clinical approaches [10]. Based on the evidence underlying the observed respiratory dysfunction in patients with NSCNP [9], a previous cohort study incorporating breathing retraining management has demonstrated improved musculoskeletal and respiratory outcomes with significant functional improvement [10]. Jafari et al. [12] reported that hypoalgesia was more evident when breathing at a lower frequency with a low inspiration/expiration ratio; however, they did not definitively present the underlying mechanisms. Diaphragmatic breathing endogenously stimulates the vagus nerve. The latter response effectively suppresses peripheral inflammatory cytokine release, decreases sympathetic tone, activates the parasympathetic nervous system’s relaxation response, and contributes to pain control [13]. We contend that the addition of breathing reeducation exercises will decrease the respiratory rate, as well as restore dysfunctional breathing and the respiratory chemistry (end tidal CO_2_ increase, pH normalization, and improved tissue oxygenation), contributing to a decrease in the excitability of the nervous and muscular system, resulting in analgesia [10]. Additionally, breathing reeducation exercises would release the dome of the diaphragm, resulting in improvement in its descent, increased rib cage mobility, and muscle tone reduction in the accessory inspiratory muscles (anterior scalene, sternocleidomastoid, upper trapezius) and the external oblique [10]. Finally, the head’s posture and the cervical spine’s mobility will improve [8].

According to the International Federation of Orthopedic Manipulative Physical Therapists (IFOMPT), manual therapy (MT) is defined as: “a specialized area of physical therapy for the management of neuro-musculoskeletal conditions, based on clinical reasoning, using highly specific treatment approaches, including manual techniques and therapeutic exercises,” considering in parallel to the psychosocial state of each patient [13]. To improve musculoskeletal and respiratory outcomes, MT stimulates a number of biomechanical, neurophysiological, and psychologically mediated mechanisms [13]. The effectiveness of MT in musculoskeletal outcomes has been examined in numerous systematic reviews [11,14,15,16]. A previous systematic review presented evidence for the effectiveness of diaphragmatic MT (DMT) in increasing rib cage, spinal, and posterior muscle chain mobility in healthy adults, as well as those with a variety of musculoskeletal and respiratory conditions [17]. 

Only one previous systematic review, which mainly addressed the association between MT, respiratory parameters, and NSCNP, could be identified in the literature [18]. However, our subsequent systematic review demonstrated the lack of existing clinical trials in examining the effectiveness of mobilization techniques of the cervical spine and diaphragm in combination with breathing reeducation exercises for NSCNP relief [19]. Moreover, it highlighted the absence of clinical trials in analyzing the effectiveness of diaphragmatic manual techniques or cervical mobilization techniques as a single intervention regaridng respiratory outcomes in patients with NSCNP [19].

### Objectives of the Proposed Study

Based on these findings and the hypotheses underlying the observed respiratory and psychological dysfunction in patients with NSCNP, the aims of this study will be to assess: (1) the effectiveness of manual therapy in the cervical spine (CMT) and diaphragm (DMT), in combination with breathing reeducation exercises (BREx), on musculoskeletal and respiratory outcomes in patients with NSCNP, and (2) the effectiveness of CMT alone or conventional physiotherapy (CP) on respiratory outcomes. Additionally, two additional objectives relating to the initial development of the outcome measures used in this study will be to examine: (3) the construct and concurrent validity of specific respiratory outcomes relating to DB in patients with NSCNP, and (4) the reliability of biomechanical-based assessments.

## 2. Materials and Methods

### 2.1. Study Design

**Figure 2:**
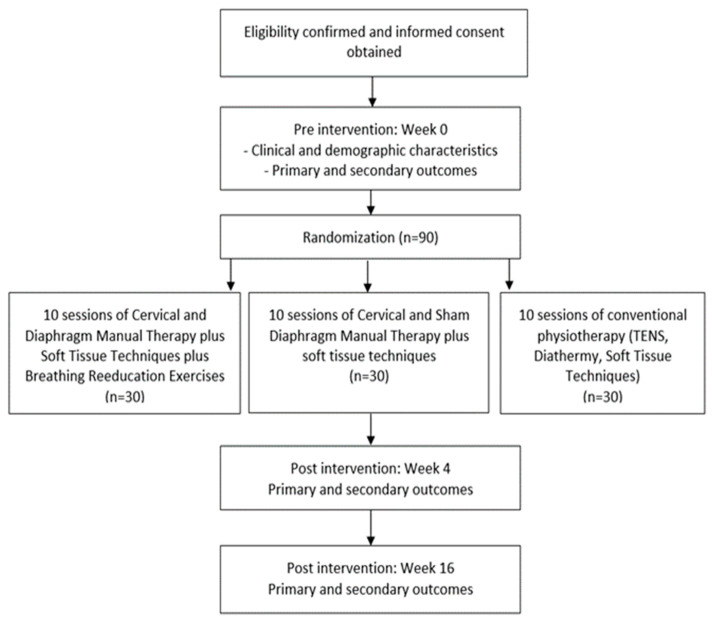
Design of the trial.

This protocol describes a randomized clinical trial (RCT) with a blinded assessor and participants, as well as the development/evaluation of several outcome measures in patients with NSCNP. We followed the SPIRIT (Standard Protocol Items: Recommendations for Interventional Trials) Statement to present this protocol [20]. The flowchart of the RCT is presented in Figure 2.

### 2.2. Ethical Approval and Registry

This study was approved by the Ethics Committee of the University of West Attica, Greece (51758—01/06/2022) under the provision that if any important protocol modifications should occur, this will be immediately communicated to the Ethics Committee. The study will be conducted according to the stipulations of the Declaration of Helsinki “Ethical Principles of Medical Research Involving Human Subjects.” The study was also prospectively registered in the ClinicalTrials.gov database (ID: NCT05229393).

### 2.3. Setting

The co-ordination of the trial will be performed by the Physiotherapy Department of the University of West Attica in Athens. Patient recruitment will be performed from several primary care orthopedic practices from areas in the southern part of Athens. Intervention and assessment will take place in a physiotherapy practice setting, also located in the southern area of Athens, Greece. All parties involved will maintain constant contact during the patient recruitment period.

### 2.4. Recruitment Procedures

The patients will be recruited from primary care orthopedic practices located near the physiotherapy practice associated with the University of West Attica for this trial, using information leaflets and posters provided at the collaborating orthopedic practices and referrals from orthopedic or spinal surgeons.

Participants will be patients with diagnosed NSCNP of a mechanical nature, referred by orthopedic or spinal surgeons for physiotherapy, provided by their public insurance, concerning their neck pain condition. Patients will contact the physiotherapy practice to book an initial appointment, where they will be informed in detail about the objectives, methods, and details of the study via relevant written material. Clarifications will be provided by the main investigator, should any questions arise. Then, if patients agree to take part, they will be provided with a consent form to read and sign.

### 2.5. Randomization and Blinding Procedures

After the initial evaluation, random allocation of participants into the trial groups will be performed. The block randomization method will be employed, using blocks of three participants, for their distribution into the following groups: Experimental Group A: Diaphragmatic Manual Therapy (DMT) plus Cervical Manual Therapy (CMT) plus Breathing Reeducation Exercises (BREx); Experimental Group B: Cervical Manual Therapy plus sham Diaphragmatic Manual techniques; or Control Group C: Conventional Physiotherapy (CP) program. Subjects will be divided into three groups by means of balanced randomization, carried out with specialized software (http://www.randomizer.org/ (accessed on 10 May 2022)). 

Individuals will be assigned in a covert manner, using sealed, sequentially numbered opaque envelopes [21]. An impartial researcher, who is not involved in the recruitment, assessment, or intervention process, will carry out the randomization and concealed allocation. The researcher in charge of carrying out the treatment programs will only open the envelopes at the time of intervention.

### 2.6. Participants and Eligibility

Eligible patients will be consecutively recruited. All eligible patients will be approached for recruitment into the study by the main researcher (physiotherapy practice owner), who will be responsible to book the initial appointment to inform patients about the trial’s aims, objectives, and methods and provide the consent form for them to sign, should they agree to participate. Then, another physiotherapist responsible for all data collection will perform all initial measurements on the same day. 

The principal researcher (PT) will initially assess each patient to establish their inclusion in the study. All patients will be required to provide written informed consent before being allowed to participate in the study, including consent to the publication of the results, and the patients will be informed that they are free to withdraw from the study whenever they like, without having any adverse effects on the provision of their treatment.

### 2.7. Participants’ Characteristics

At least 90 adult volunteers of both genders, aged between 25 and 65 years, and with mechanical chronic neck pain (for more than 12 weeks), Grade I or II according to the Task Force on Neck Pain classification (Nordin 2009) [22], negative Spurling’s test, traction test, upper limb tension test, and shoulder abduction test, understanding and fluently speaking the Greek language, and the absence of another serious pathology, will be included in the present study. The diagnosis of non-specific mechanical chronic neck pain will be performed after history taking and physical examination (according to the clinical protocols of IFOMPT) [23] and medical X-rays, according to the Canadian Cervical Spine Rule (C-Spine) and the National Emergency X-Radiography Utilization Study (NEXUS) [24]. Patients will also be selected on the basis of demonstrating DB in at least one of a series of tests conducted to assess the extent of their DB (biomechanical, biochemical, psychological).

The following criteria will be used to determine who is excluded: history of cervical trauma, whiplash injury, or cervical surgery; cervical hernia; spinal degenerative disease; upper limb pain radiation; having received physiotherapeutic treatment for the cervical region within the previous three months; metabolic bone diseases and osteoporosis; cardiac or respiratory insufficiency; pregnancy; neoplasm; infectious diseases; psychological diseases; red flags (overnight pain, serious muscle spasm, weight-loss unrelated to diet, daily headache, dyspnea, confusion, and loss of consciousness); recent consumption of analgesics, anti-inflammatories, or muscle relaxants; systemic illnesses; and fibromyalgia.

### 2.8. Initial Assessments

**Figure 3:**
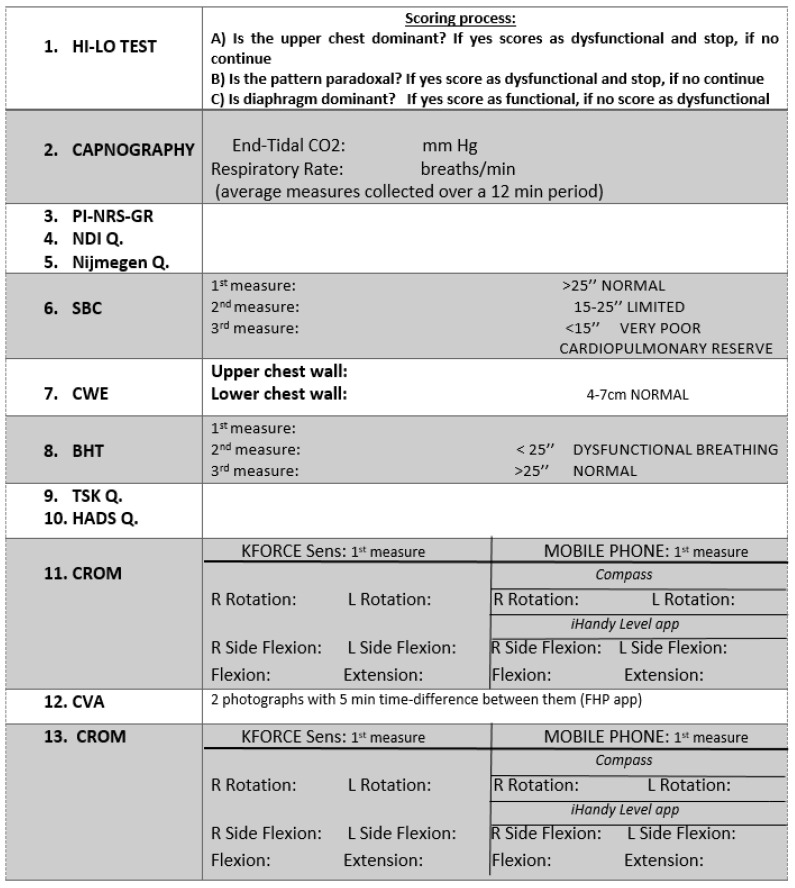
Order of outcome assessments.

Initially, demographic and past medical history details (age, height, body mass, medical history, previous illnesses, concomitant diseases, use of medications, prior surgeries, physio therapeutic treatment, professional occupation and other aspects) will be collected, and a series of special tests will be administered by a physiotherapist, who will be blinded to participants’ subsequent group allocation. A physiotherapist with a Master of Science in Advanced Physiotherapy and at least six years of experience in clinical practice will evaluate the assessment of all outcomes. The planned sequence of outcome assessments is depicted in Figure 3. 

### 2.9. Outcomes

The evaluations will take place at all time-points: before the treatment sessions (week 0), after ten treatment sessions (week 4), and four months after the beginning of the trial (week 16). The primary outcomes will be: functional disability (NDI) [25] and pain (VAS) [26]. Several secondary outcomes will also be evaluated: cervical range of motion (CROM) [27], craniovertebral angle (CVA) [28], Nijmegen Questionnaire (NQ) [29,30], Hi-Lo test [31], breath holding time (BHT) [32,33], single breath count (SBC) [34], end tidal CO_2_ (ETCO_2_) [35] and respiratory rate (RR) [36], chest wall expansion (CWE) [37], the Hospital and Anxiety Depression Scale (HADS) [38], the Tampa Scale for Kinesiophobia (TSK) [39] and adverse events [40]. All outcomes are briefly presented below. The corresponding references for each of the above outcomes include reliability and validity details, except for some further testing proposed in Section 2.10 and Section 2.11.

#### 2.9.1. Primary Outcome Measures

The Neck Disability Index (NDI) [25]: The NDI is a disability self-report tool in relation to neck pain. It consists of 10 questions, with 6 alternative responses each, that relate to the ease of performing different tasks, pain severity, and the coexistence of headache. Each item’s score ranges from 0 (no pain and no functional limitation) to 5 (worst pain and maximum limitation), for a total score that ranges from 0 (no disability) to 50 (totally disabled).

Pain Intensity-Visual Analog Scale (VAS) [26]: Subjective pain intensity registration, by the selection of a value between 0 (no pain) and 100 (maximal pain) that best describes the intensity of pain felt in the last 24 h.

#### 2.9.2. Secondary Outcome Measures

Range of motion (ROM) [27]: A smartphone-based application (iHandy Level: https://play.google.com/store/apps/details?id=com.ihandysoft.carpenter.level.free&hl=et&gl=US (accessed on 10 May 2022)) downloaded to a smartphone (Model P30/ELE-L29, Huawei Smartphone, China 2019, purchased January 2021), in parallel with a sensor-based commercially available device (KFORCE SENS electronic goniometer, KINVENT-France), will be used to accurately measure ROM during neck movements of flexion-extension, left-right side flexion, and left-right rotation. 

Craniovertebral Angle (CVA) [28]: Lateral photography examination of the FHP through lateral photographs can provide very reliable estimates. FHP will be estimated in a relaxed sitting position for all participants. A smartphone-based application (Forward Head Posture-FHP app), available on Google Play (https://play.google.com/store/apps/details?id=com.ysjworld.fhp&hl=en&gl=US (accessed on 10 May 2022)), will be used for CVA angle measurements.

Nijmegen Questionnaire (NQ) (Greek version 2014) [29]: Screening tool used to detect patients with hyperventilation complaints and DB patterns. Scores > 19 are used as the cut-off score to separate hyperventilatory patients from normal subjects [30]. NQ values in healthy individuals range from 10 to 12 ± 7, and values have been observed to decrease towards these levels in subjects with DB after breathing retraining [30].

Hi-Lo test [31]: The examiner will be given instructions on how to conduct and record results from the Hi-Lo test, including the following: “Examiner at the front and slightly to the side of the individual has to place one hand on the patient’s sternum and the other hand on their upper belly. The examiner must evaluate if and to what extent abdominal or thoracic motion predominates while breathing. Additionally, paradoxical breathing needs to be considered, as well as the degree of abdominal or thoracic breathing rate.” This test evaluates DB.

Breath Holding Time (BHT) [32]: This test is an indicator of a person’s respiratory response to biochemical, biomechanical, and psychological factors, and it seems that abnormally shortened BHT may indicate abnormalities in respiratory function that are closely related to DB. Participants will be instructed to assume a comfortable sitting position and breathe normally and gently, in and out, and at the end of a normal exhalation, they will be asked to pinch their nose and hold their breath. The instruction is to hold their breath to the point when they cannot hold their breath any longer and are required to breathe in again. According to Kiesel (2020) [33], a BHT < 25 s is considered as a sign of DB.

Single Breath Count (SBC) [34]: Participants will be requested to count out loud after maximal inspiration. The ability to reach a count of 50 (corresponding to 25”) is considered as normal respiratory function. This test evaluates DB.

End-Tidal CO_2_ (ETCO_2_) [35] and Respiratory Rate (RR) [36]: These outcomes are measured by a standard capnograph unit. An ETCO_2_ average value that is less than 35 mm Hg over a 12 min period and an average RR of more than 16 breaths/min, will be considered as signs of DB.

Chest Wall Expansion (CWE) [37]: A tape measure will be used to determine the difference between the readings of thoracic cage circumference acquired during deep inspiration and expiration, with higher values indicating a better result.

Hospital and Anxiety Depression Scale (HADS) (Greek version 2008) [38]: Anxiety and depression are measured by the anxiety and depression level assessment scale commonly used in musculoskeletal research, with 7 items contained in each of the subscales (range of scores between 0–21 for each), assessing the two constructs. For each of the two subscales, a score of >8 denotes clinically relevant anxiety/depression.

Tampa Scale for Kinesiophobia (TSK) (Greek version 2005) [39]: Kinesiophobia is a frequently utilized psychological measurement of pain-related fear in musculoskeletal clinical conditions. The translated version of the original 17-item questionnaire will be used.

Adverse events [40]: Potential adverse effects of therapy will be evaluated during and after the physiotherapy treatment, according to a proposed 5-level rating. Should any patient present with serious adverse events, they will be withdrawn from the trial and referred back to their doctor for further evaluation.

The order of administration of the outcome assessments is presented in Figure 3. Any reactive or interactive effects of the measurements [21] conducted sequentially may not be avoided; however, these effects are expected to affect participants allocated in either of the treatment groups in the same manner. All outcomes will be accumulated for statistical analysis, which will be conducted by a professional statistician.

### 2.10. Respiratory Outcomes Validity Investigation in Patients with NSCNP

#### 2.10.1. Construct Validity of the NQ 

The assessment of the construct validity of the NQ will be performed via exploratory factor analysis, similar to its previous validation in a population with asthma [29], to examine the factor structure of the NQ in a population with NSCNP. This study has not been performed in a population with chronic pain and without a respiratory condition, such as the one under study.

#### 2.10.2. Concurrent Validity of Respiratory Outcomes Assessing DB

The examination of relationships between the following respiratory outcomes of DB (concurrent validity) will be performed: the NQ (psychophysiological dimension), ETCO_2_ (biochemical dimension), and RR, Hi-Lo, BHT, SBC, CVA, CWE (biomechanical dimension). Similar research has previously been conducted [41], but not in a population with NSCNP.

#### 2.10.3. Prevalence of DB in Patients with NSCNP

As there are no established reference standards for DB [33], the diagnostic accuracy of respiratory measurements relating to DB in patients with NSCNP cannot be examined. Instead, as previously proposed [42], the prevalence of DB in patients with NSCNP can be presented, using cut-off scores established from previous studies performed in an asymptomatic population without a respiratory condition (ETCO_2_ with a cut-off score of 35 mm Hg, the NQ with a cut-off score of 19), and the Hi-Lo test, for the determination of the type of breathing, as single or combined (in pairs, or using all three) outcomes, to identify the percentage of the population with NSCNP that exhibits respiratory dysfunction. 

### 2.11. Biomechanical Outcomes Reliability Investigation in Patients with NSCNP

#### Reliability of Cervical ROM and FHP

As novel measurement methods will be used to measure cervical ROM and CVA, the reliability of both proposed cervical ROM measurement methods will be separately evaluated, and parallel forms reliability between the two methods will be tested in a proportion of this population. Additionally, CVA reliability will be measured, and this will be conducted with a new smartphone-based application. All test-retest reliability measurements will be performed by a single examiner on the same day of initial assessment, with adequate times between measurements (Figure 3). All reliability assessments will be performed in the first 35–40 participants of the main trial to assess their reliability level. Pre-post comparisons of any of these measurements that prove not to be sufficiently reliable will not be further considered. 

### 2.12. Interventions

Ten treatment sessions will be administered to all participants, thrice for the first two weeks and twice for the third and fourth weeks, lasting for 40 min per session. The sessions will be individually administered in a reserved room with adequate lighting, music, and air conditioning. 

The manual therapy program will be applied by a physical therapist (main researcher), a clinical instructor of manual therapy, PhD candidate, with an OMT diploma from a recognized IFOMPT program, certified as Mulligan Practitioner, with two Master of Science degrees from the Athens Medical School, Greece, and 20 years of experience in clinical practice. A physical therapist with a Master of Science degree from the Athens Medical School and 17 years of experience in clinical practice will administer the traditional physiotherapy program (Group C) and the sham diaphragmatic manual therapy component (Group B). Sham diaphragmatic manual therapy will be administered via a therapeutic ultrasound device [43]. 

The participants can only receive the assigned treatment. They will be advised against combining the treatment they were allocated to with medication or other physical therapy approaches. Any deviation from the treatment sessions administration (missing more than one appointment) or any worsening of symptoms will be reasons for exclusion. The interventions proposed for each group are described briefly below, and in detail in the Supplementary Materials. All selected interventions are widely applied in the field of physical therapy and are safe when administered by appropriately trained physical therapists. 

**Group A:** This group will receive CMT [44,45,46,47], DMT [48,49,50], and BREx [51,52]. Initially, cervical manual therapy (CMT) will be applied for 20 min, consisting of cervical spine inter-vertebral mobilization techniques, according to the Mulligan Concept, and soft tissue techniques. Subsequently, in order to indirectly stretch and mobilize the fibers of the diaphragm, diaphragmatic manual therapy will be used, which should result in improved muscle contraction and reduced tension. The doming diaphragmatic technique and the manual diaphragmatic release technique, both as described by Leon Chaitow, will be among the experimental techniques employed. For 10 min, both maneuvers will be performed in 2 sets of 10 repetitions each, separated by 1 min. Finally, breathing reeducation exercises, according to the Papworth method, will be implemented for 10 min, consisting of: (i) recognition of the abnormal breathing pattern, and practicing stomach and nose breathing retraining, (ii) slow breathing and controlled breath holding, (iii) relaxation techniques training, (iv) progressing breathing retraining in everyday life, with emphasis on postural correction, and (v) breathing reeducation exercises for home use, with the help of a booklet and videos, with emphasis on postural correction and with the instruction to practice these at least once per day. 

**Group B:** This group will receive CMT/soft tissue therapeutic techniques, plus sham DMT. Initially, the same manual therapy for the cervical spine (according to the Mulligan Concept and soft tissue techniques) as that used in Experimental Group A will be administered for 20 min. Additionally, sham DMT will be applied via therapeutic ultrasound (Sonopuls 692 Enraf-Nonius) sham administration in the sitting position for 10 min [38]. The equipment will be switched on, but the intensity will not be raised (0 W/cm^2^); therefore, the equipment will appear working, but no therapeutic dosage will be administered. The same therapist will carry out all the sham ultrasound techniques. Moreover, for 10 min, guidelines for activities of daily living (housekeeping: cleaning, sweeping) will also be provided, with emphasis on postural correction.

**Group C:** This group will receive a typical conventional physiotherapy program, therefore acting as an active control group for this trial. Initially, Transcutaneous Electric Nerve Stimulation (TENS) will be applied, with a pulse duration of 250 microseconds at a frequency of 80 Hz for 15 min in the suboccipital region and the trapezius bilaterally. Then, Pulsed Microwave Diathermy (Enraf-Nonius RADARMED 950+), will be applied in the sitting position, for 10 min. Soft tissue techniques (deep soft tissue massage) will be applied at a slow speed for 15 min. Massage therapy, included gliding and kneading techniques will be applied over the trapezius (upper, lower, and middle fibers), splenius capitis, and levator scapulae muscles [53].

At the end of the interventions, leaflets will be given to the participants of Group B and Group C, describing general stretching exercises for the cervical spine.

Further details of the techniques applied are provided in the Supplementary Materials. However, leaflets with intervention details will be provided to participants of all groups to enhance adherence to the program, as the exercises will also be performed by participants at home. 

At the end of the trial, subjects will be asked which group they thought they were allocated in, to determine any potential bias.

### 2.13. Anticipated Dates of Trial Commencement and Completion

The trial is scheduled to commence in October 2022, and completion is scheduled for June 2023. Currently, the RCT has not yet started.

### 2.14. Sample Size Calculation

The estimation of the sample size was performed with the use of the G*Power, version 3.1 program. For a three parallel groups design involving three repeated measures (pre, post-treatment, and one follow-up), in order to achieve a statistical power of 0.80 in detecting a medium effect size (Cohen’s f = 0.25) in the interaction effect, at a significance level of 5% (0.05), it was calculated that a minimum sample size of 80 individuals would be required. Taking into consideration a dropout rate of 10%, it was estimated that 30 individuals per group would need to be initially recruited [21].

### 2.15. Statistical Analysis

Baseline characteristics and all outcome measurements will be analyzed initially in each group for normality of their distribution using the Shapiro–Wilk Test. Descriptive statistics will be used to present the distribution of the data.

Statistical analysis for the RCT will be conducted, based on intention-to-treat analysis. In this way, the individuals will be analyzed in the groups in which they were randomly allocated. A separate analysis will be conducted in case any protocol deviations occur for part of the population, such as low adherence with the organized treatment sessions, low home compliance (<50% attendance), or should patients withdraw themselves from the RCT. Linear mixed models (two-way mixed ANOVA) with one repeated measures factor (time) and another between-groups factor (group), especially considering the interaction between the factors of time (week 0, week 4, and week 16) and group (A and B, A and C, B and C). Additionally, post hoc tests will be employed to determine the source of the interaction, if significant. 

The principal components analysis method will be performed for the factor analysis of the NQ. Pearson’s bivariate correlations will be employed for the examination of concurrent validity of respiratory outcomes.

For all analyses, a significance level of 5% will be considered. All data processing will be performed using the IBM SPSS software, version 28. 

## 3. Discussion

Previous research has investigated many interventions and outcomes for the amelioration of symptoms in NSCNP; however, until now, many patients have reached a plateau in their rehabilitation, without full resolution of their symptoms [6,7]. One explanation for this partially successful approach is that patients with NSCNP present not only musculoskeletal, but also respiratory disorders, both contributing to the symptomatology of chronic neck pain [8,9,10]. 

The present study’s innovative approach resides in its emphasis the importance of recognizing all components of breathing dysfunction. DB is multi-dimensional and contains biochemical, biomechanical, and psychophysiological components [33,42]. The present study surmises that no single test or questionnaire can identify breathing dysfunction; therefore, a battery of tests is proposed for the identification of breathing dysfunction [30,31,32,33,34,35,36]. As a result, additional validity investigations and interventions are required in patients with NSCNP in order to cover additional aspects of their pathology.

The study aspires to provide a more holistic approach in patients with a condition which has, up until now, been considered to involve mainly the neuromusculoskeletal system [18,19]. The specific components of this approach have not yet been determined. It is possible that the release of the diaphragm, in combination with breathing reeducation, will not only decrease pain and other musculoskeletal-related outcomes, but also may improve the body’s ability to achieve homeostasis, in general [19]. 

There are certain limitations in the study that must be taken into account. The physiotherapist in charge of carrying out the treatment cannot be blinded, due to the nature of the proposed therapeutic procedures. The intervention’s length, frequency, and number of sessions is similar to the one generally provided in current practice, although it may be that longer-term interventions or a different components’ combination can be even more effective. Finally, no true control group will be included; instead an active control (conventional physiotherapy, CP) group is selected for ethical reasons, as well as for blinding purposes, as in this manner, all patients will receive an active treatment for their conditions [21]. 

## 4. Conclusions

This randomized controlled trial will determine the effectiveness of cervical and diaphragmatic manual therapy, plus breathing reeducation exercises, in NSCNP patients. The present study is expected to have high internal validity, since this protocol is planned according to accepted methodology regarding the randomization, concealed allocation, blinding of examiners, intention-to-treat analysis, and appropriate sample size calculation. This study is innovative, as it combines the mobilization of the cervical spine with diaphragmatic mobilization and breathing reeducation exercises, to address the rehabilitation of patients with NSCNP in a more comprehensive manner. 

## Data Availability

Not applicable.

## Supplementary Materials

The following supporting information can be downloaded at: https://www.mdpi.com/article/10.3390/diagnostics12112690/s1, Table S1: Schedule of enrollment, intervention, and assessments; Details of therapeutic interventions. References [54,55,56] are cited in the supplementary materials.
