# Supplementary material for: The Effectiveness of Manual Therapy in the Cervical Spine and Diaphragm, in Combination with Breathing Reeducation Exercises, in Patients with Non-Specific Chronic Neck Pain: Protocol for Development of Outcome Measures and a Randomized Controlled Trial
Source: Diagnostics (Basel). 2022 Nov 4;12(11):2690. doi: 10.3390/diagnostics12112690 (PMC9689657; doi:10.3390/diagnostics12112690)
Supplement: Supplementary file 1 [file diagnostics-12-02690-s001.zip › diagnostics-1936124-Intervention details.pdf]

Supplementary Materials

Details of Therapeutic Interventions

Experimental Group A: Cervical Manual Therapy (CMT)/soft tissue techniques + Diaphragmatic Manual Therapy (DMT) + Breathing Reeducation Exercises (BREx) Program

A) CMT will be applied for 20 minutes and consists of cervical vertebrae mobilization techniques, according to the Mulligan Concept, and soft tissue techniques, following patient examination based on clinical reasoning [1, 2, 3, 4]. Specifically, the following techniques will be applied:

i) **NAGs** (natural apophyseal glides) (Figure S1) are passive oscillatory mobilizations that will be applied as the first-choice intervention in that group between the C2 (2nd cervical) and C7 (7th cervical) vertebrae. Patients will be asked to sit and rest their back against a chair, and a pillow placed in front of their chest will support their arms for reducing the tension in the neural and myofascial tissues around the neck and scapula area. The therapist will stand at the left side of the patient and will stabilize the head of the patient with his left shoulder and his chest, in a neutral head position (without side flexion or rotation). The middle phalanx of the left little finger will be placed around the spinous process (bilateral glide) or the articular pillar (unilateral glide) of the vertebra above the joint (intervertebral level) that must be mobilized. The other fingers will wrap around the remaining above-situated cervical spine vertebrae and occiput. Then, from a slight neck flexion position of the cervical spine, a light distraction will be applied for better contact of the therapist's hands. The lateral border of the thenar eminence of the therapist's right hand will be placed slightly over the little finger of the therapist's left hand. A glide along the treatment plane (cervical spine facet plane), in a cranial direction and towards the eyes of the participant, will be

applied by pushing upward and forward with the therapist's right hand (mobilizing hand) via the therapist's little finger of the left hand. The mobilization will be applied via rhythmical oscillatory movements (1-2 per second), and no more than 6 repetitions will be applied.

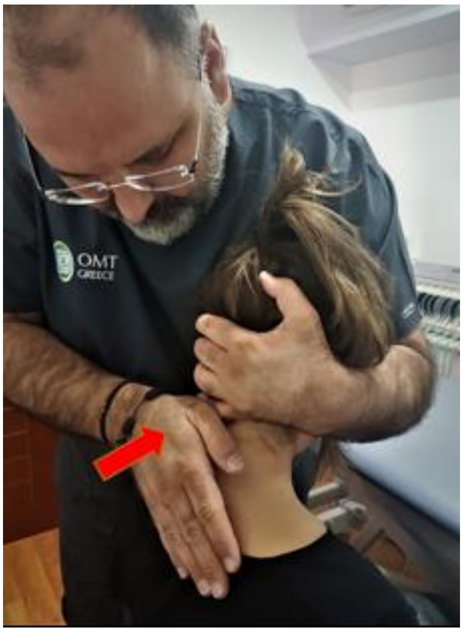

Figure S1

ii) **Reverse NAGs** (reverse natural apophyseal glides) (Figure S2) are passive oscillatory mobilizations applied at the lower cervical and upper thoracic spine. The patient will be seated, and the therapist will be standing next to the patient. The therapist's forearm will cradle the patient's head to his body, and the therapist will place their 5th (little) finger across the posterior part of the vertebra above the identified painful segment. With his thumb and index finger, the therapist will form a "v" and make contact with the articular pillar of the lower cervical spine or transverse processes of the upper thoracic vertebra. A mobilization will take place when the therapist glides the superior facets of the lower vertebra up on the inferior facets of the above-situated vertebra, cranially and towards the eyes of the participant, along the treatment plane.

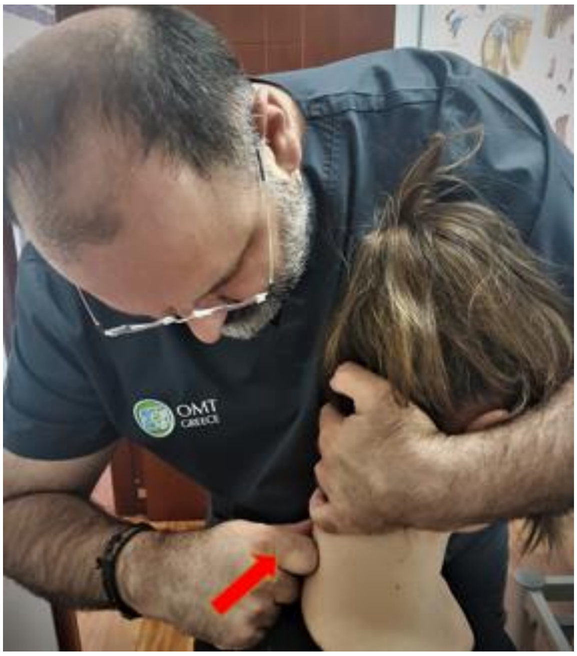

Figure S2

iii) **SNAGs** (sustained natural apophyseal glides) (Figure S3), are a combination of a sustained unilateral facet glide with movement. Load-bearing positions will be selected and will be performed at each cervical spine level. The technique must be executed without pain at the end of the joint movement. With the patients in a seated position, cervical SNAGs will be applied with the thumb of the one hand supported by the thumb of the other hand, placed, depending on the indication, on either the articular pillar or the spinous process of the upper vertebra of a certain intervertebral level. The therapist will apply a passive intervertebral movement in a superoanterior direction, along the facet plane (in a direction towards the patient's eyes). The therapist will maintain this glide as the patient actively moves the cervical spine in any range of restricted physiological movement and then sustain it at the end range position for a few seconds. When the patient returns to the starting position of the active movement, the glide will be released. For 3 weeks, this mobilization will be repeated 6 times per session.

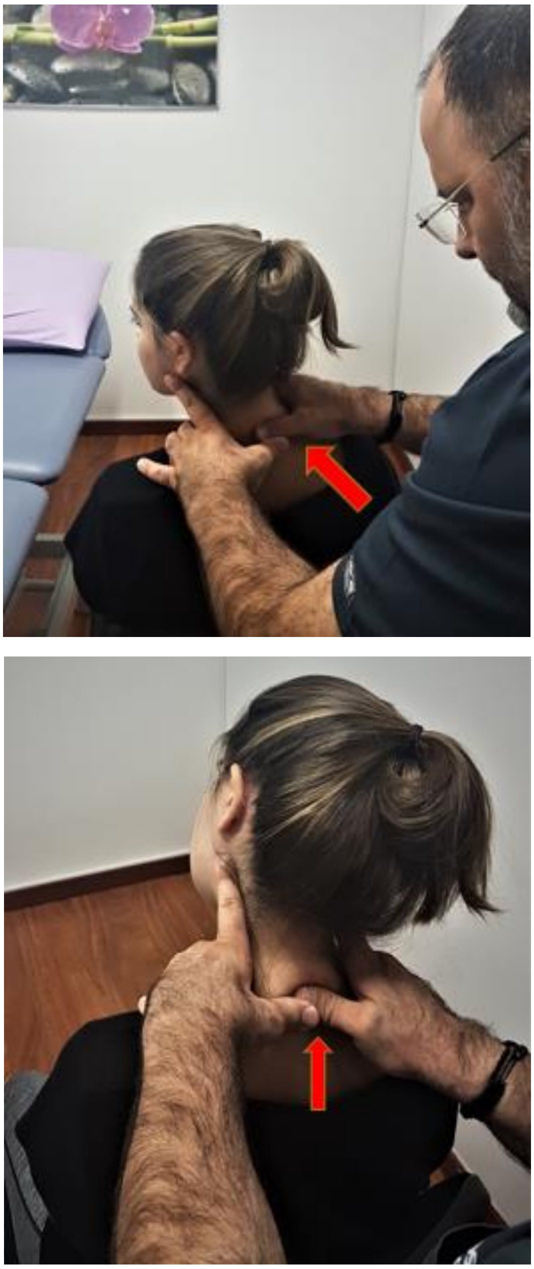

Figure S3

iv) **Traction technique**

The patient will lie supine, with a small pillow under the head. The therapist will place the lower part of his dominant forearm under the patient's cervical spine so that the ventral border of the radius is inserted under the base of the patient's occiput. The index and middle fingers of the other hand will be placed under the patient's chin. Traction will be applied as the therapist's forearm pronates, and an

equal and simultaneous pressure will be applied under the patient's chin. According to the Mulligan Concept, traction is applied in the upper cervical spine, but according to Kaltenborn, a generalized traction is applied to the entire cervical spine. The therapist will maintain the traction for at least 10 seconds.

v) **Soft tissue techniques**

Treatment methods for such dysfunction include post-isometric muscle relaxation (PIR) and muscle energy techniques (MET), in combination with techniques that affect the postural muscles, such as eye movements, respiratory synkinesis (inhalation-exhalation), and reciprocal inhibition (RI).

The muscle fibers that need to be relaxed must first be brought into a position in which they attain their maximum length without stretching, taking up the slack in the same way as in joint mobilization. A minimum isometric force (about 25-30% of available force) will be applied, and the patient will be asked to resist. This resistance will be held between 10 and 30 seconds, and then the therapist will ask the patient to relax completely for a period between 15-30 seconds. The procedure will be repeated three to five times. If required, PIR will be combined with methods that affect the postural musculature collectively, such as resistance to the antagonist muscles for one repetition (reciprocal inhibition) (RI), or with eye movements, especially for treating muscles such as the scaleni and the sternocleidomastoid (usually, eye movement facilitates movements of the head and spine, in the direction of the gaze, and inhibits movements in the opposite direction), or with respiratory synkinesis (inhalation, exhalation), in which movement in one direction is associated with inhalation, and in the opposite direction with exhalation. Usually, inhalation has a facilitating effect (combined with isometric resistance) and exhalation an inhibitory effect

(combined with relaxation), especially on the muscles of the trunk. All soft tissue techniques will be performed according to the Chaitow (2014) [5] and Lewit (2010) [6] methods.

B) Subsequently, **Diaphragmatic Manual Therapy (DMT)** will be carried out. This consists of techniques intended to directly or indirectly stretch and mobilize the diaphragmatic muscle fibers. This will help to improve muscle contraction and decrease tension. The maneuvers include the Doming Diaphragmatic Technique, as described by Leon Chaitow [5] and Karel Lewit [6], and the Manual Diaphragmatic Release Technique, as described by Leon Chaitow [5]. Both maneuvers will be performed in two sets of 10 repetitions, within a 1-minute interval, for 10 minutes [7].

Trigger Points (TrPs) Approach Method

Each subject will be positioned in an upright sitting position, but not slumped. The therapist will stand behind the patient, supporting the patient's trunk, and pass their hands carefully around the thoracic cage, introducing fingers under the costal margins, close to the diaphragm's attachment (Figure S4). If TrPs are present, marked resistance will be felt and the patient will experience some pain.

The technique that the therapist will follow is PIR. The therapist will ask the patient to breathe in a little. Then, with a closed mouth, the patient will be asked to pinch their nose and breathe in against isometric resistance for 5–10 seconds, and then breathe out slowly. He will be able to achieve this because he has breathed in a little at the beginning. The patient will learn to perform isometric resistance, not by pinching his nose, but by closing his glottis, as when pronouncing the consonant 'K.' After two or three repeats the patient will have to perform reciprocal inhibition (RI) by actively breathing out as far as possible.

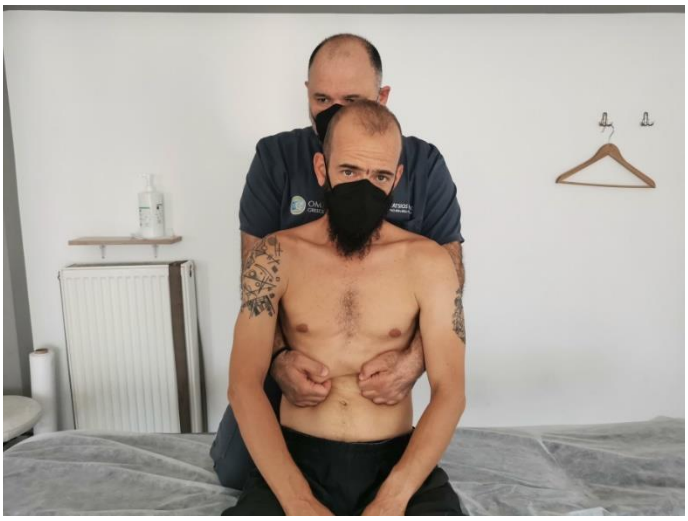

Figure S4

Indirect Approach Method (Positional Release Technique)

The therapist will passively flex the patient's trunk to then carefully determine which direction offers the greatest degree of freedom and ease of motion. The therapist will have to maintain the trunk of the patient towards the rotation direction that feels looser (having more range and a softer end-feel) and, in this position, the therapist's hands have to support and follow the tissues as a slow releasing, unwinding process ensues. When the diaphragm resumes a free rhythmic vertical motion, the rotational preference should be retested, and should be more symmetrical after the application of the technique.

Direct Approach Method (Stretching Technique)

The same starting position, is used, with the practitioner standing behind the seated patient, fingers curled under the costal margins. The practitioner gently flexes the trunk of the patient in order to relax the rectus abdominis. When the patient exhales, the therapist will grasp the lower ribs and costal margins and will ease their hands caudally, causing a stretching effect (lengthening) of the diaphragm. This firm, but gentle traction is maintained as the patient inhales. The maneuver will be

performed in three sets of 10 breaths, with a 1-minute interval between them [5].

C) Finally, **breathing reeducation exercises,** according to the Papworth method [8-10], will be implemented for 10 minutes. The aim of all breathing reeducation techniques and exercises is the limitation-restriction of hyperventilation, hypocapnia, and dysfunctional breathing, symptoms that patients with non-specific chronic neck pain usually present with. The first step is identifying and inhibiting an abnormal upper thoracic respiratory pattern, reeducating diaphragmatic and slow breathing through the nose, and introducing brief respiratory pauses after each exhalation. Secondly, breathing exercises aim to maintain the natural position and alignment of the spine through the reeducation of normal breathing pattern and diaphragmatic release. The phases of breathing intervention will consist of:

i) Assessment/recognition of the abnormal breathing pattern, and practice of diaphragmatic and nose breathing retraining;
ii) Slow breathing and controlled breath holding;
iii) Training in relaxation techniques;
iv) Integrating and progressing breathing retraining in everyday life;
v) Home-based breathing reeducation exercises, with the help of a booklet and videos, with the instruction to practice at least once per day.

i) Assessment/Recognition of the abnormal breathing pattern, and practice of diaphragmatic and nose breathing retraining,

First step

Participant position

- Half-lying supine in a comfortable position (~45˚flexion of the spine from the neutral supine position).

- o The participant must have a comfortable, but not slouched position.
- o A pillow is necessary underneath the head and knees.

Instructions to participant (Figure S5)

- o Place one relaxed hand over your stomach and the other on the upper chest and clavicle.
- o Breath normally and slowly, as normal.
- o Take a deep breath.

Notification

- o The therapist must notify the patient if they breathe through the mouth or nose.
- o If the patient is hyperinflating the thorax through the upper chest first, with little or no low-chest inflation, or even drawing in the stomach at the peak of inspiration, the therapist must notify them of this.

Patient recognition

- o Through using their hands, the patients must to recognize the difference between forceful (“big”) and softer (“quiet”) breathing.
- o The patient must feel the movement of their upper chest or their stomach while they breath [6].

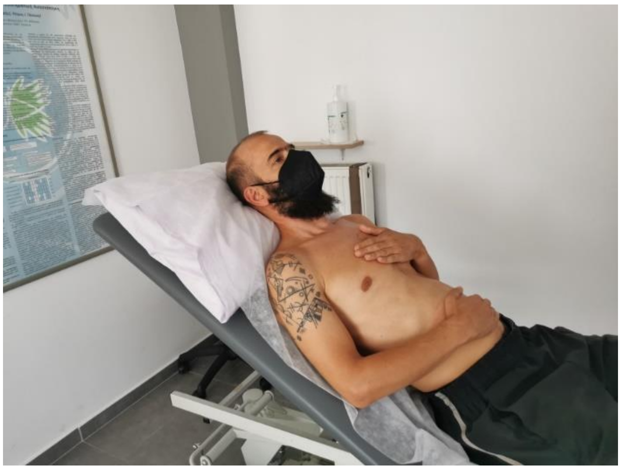

Figure S5

Second Step

Participant position

- o Half-lying supine in a comfortable position (~45˚flexion of the spine from the completely supine position).
- o One or two pillows should be behind the head and knees.

- One relaxed hand should be over stomach and the other on upper chest and clavicle.

Instructions to participant

- Close your mouth and try to breathe easily, slowly, and softly through your nose.
- Be careful not to inhale with "big" breaths.
- Now, try to raise only the stomach during inhalation.
- Exhalation is passive through nose.
- Let your stomach fall gently.

Additional instructions to participant

- If you want to yawn, cough, or sigh, try to do it with your mouth closed.
- Practice stomach nose breathing regularly during the day.
- Inform patient about the positive effect of nose breathing.

Practice at home

- Twice a day, practice the new breathing pattern, through the nose, for 10 minutes and increase this progressively.
- Use a mirror while practicing to obtain feedback.
- Practice breathing in different positions (sitting with slight flexion of the trunk 45˚) [8, 10].

ii) Slow breathing and controlled breath holding with emphasis on stress management

<u>First step (Slow Breathing)</u>

Participant position

- Half-lying in a supine, comfortable position (~45˚flexion of the spine from the completely supine position).
- One or two pillows should be placed behind the head and knees.
- One relaxed hand should be placed over stomach, and the other on the upper chest and clavicle.

Instructions to participant

- Close your mouth and try to breathe easily, slowly, and softly through your nose.
- Relax your shoulder, chest, jaw, and facial muscles.

- Try to slow down your breaths. Sometimes, it helps to count in your head while you are breathing, e.g., breathe in for a slow count of 2, and breathe out for a slow count of 3.

Additional instructions to participant

- It is essential for you not to pause for long because they will feel unpleasant or faint. Record your practice sessions on a calendar.
- As it gets easier, you can try to increase the length of time that you pause at the end of your exhalation, before taking the next breath.
- When you are able to breath easily, slowly, and softly through the nose, without the feeling "breath hunger" or other unpleasant feelings, you should try to decrease the number of breaths by lengthening the time of the pause. You should practice this for 1-2 minutes and up to 15 minutes. According to Jafari et al (2020) [11], analgesia is achieved at six breaths per minute. Twice a day, practice slow breathing progressively from 1 or 2 minutes up to 15 minutes.

Second step (Controlled Breath Holding)

This technique aims for the patient to be able to hold his breath without feeling "breath hunger." As the patient gets used to breath holding, they will be able not to feel breathless in everyday life.

Participant position

- Sitting position and well supported.
- Comfortable and relaxed shoulders and chest.

Instructions to participant (Figure S6)

- Breath in and out using the diaphragm (inflating the stomach), normally and gently through the nose.
- At the end of exhalation, pinch your nose and hold your breath.
- Keep holding your breath until you feel the desire to inhale. Then release your nose and breathe normally.
- Once you are able to hold your breath in this way, do 3 repetitions, with a 1-minute rest in between repetitions.

Additional instructions to participant

- If you feel hungry for breath, then you may have held your breath for too long.
- Try to progressively increase the holding time.

- A calendar or a smartwatch is necessary. You may register the exact position you used when they did the exercise, the time, the day, the repetitions, and the duration of exercise.

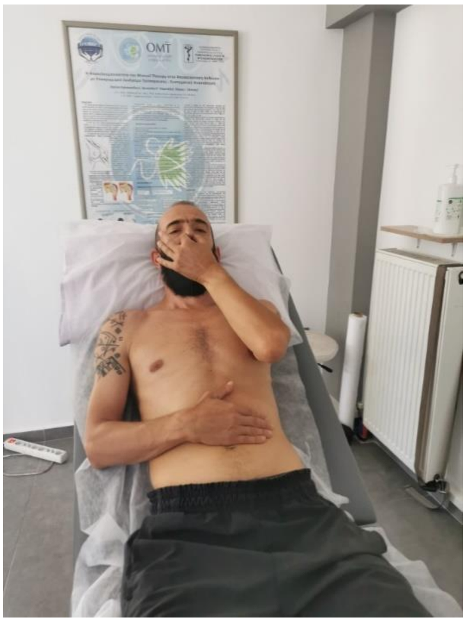

Figure S6

Practice at home

- Twice a day, practice controlled breath holding, 3 repetitions, with a 1-minute rest in between repetitions.

iii) Relaxation techniques training

The aim of those techniques is for the patient to be able to relax the whole body.

- Breathing

Close your eyes inhale and exhale through your nose using your diaphragmatic breathing.

- Feet

Gently, plantar flex your toes towards the floor or away from your body; hold this position for the count of 5 seconds and relax. Repeat once.

- Legs

Contract your thigh muscles and push your knees down; hold for 5 seconds, and relax. Repeat once.

- Buttocks

Contract your buttocks and hold for 5 seconds. Relax and repeat once.

- Back/abdomen

Push your back into the floor/bed; hold for 5 seconds, and relax or if you are in sitting position; pull your stomach muscles in, hold for 5 seconds and relax. Repeat once.

- Shoulders

Shrug your shoulders towards your ears, hold for 5 seconds and relax. Repeat once.

- Arms

Push your arms downwards; hold for 5 seconds and relax. Repeat once.

- Hands

Clench your fists; hold for 5 seconds and relax. Repeat once.

- Eyebrows

Raise your eyebrows as though surprised; hold for 5 seconds and relax. Repeat once.

- Jaw

Keeping your mouth closed gently stretch your jaw downwards; hold for 5 seconds and relax. Repeat once.

- Head

Push your head down into the pillow. Hold for 5 seconds and relax. Repeat once.

Allow yourself to relax your whole body now, and notice how your body and muscles feel. Stay in the same position for another 5 minutes and focus on your gentle breathing pattern. You can practice this relaxation technique at home once per day.

iv) Progressing breathing Retraining in everyday life

The aim of this stage is to integrate diaphragm and slow nose breathing in everyday life, physical activities (speaking, swimming, walking, gardening etc.), social activities (playing with children or pets), and work-related activities (dealing with work stress).

Instructions to the patient

- Try to breath in and out slowly and through nose, even under the most difficult conditions.
- Over time, you will be able to recognize faulty breathing patterns and you will be able to correct them.
- Apply a slow, through the nose, diaphragmatic breathing pattern at speech (max 4-6 words spoken during slow exhalation, with breathing pauses, and slow inhalations), at temporary alleviation of cough (pauses till tickle on the neck stops), at yawning, and at sighing (closed mouth).
- Try to use a calendar or a mobile phone reminder for the implementation of diaphragmatic slow nose breathing pattern and breathing exercises [12].

At the begging of every session, each participant will be assessed for their compliance in relation to the exercises (use of calendar or via questions about their exercise routine between-sessions) and the effective performance of every exercise. In case a patient had not comprehended or could not perform the exercises correctly, the previous session will have to be repeated.

After the end of the 10 sessions, participants will follow the instructions presented in an exercise and advice sheet. With the aim to improve patient compliance, and at 4th and 8th week, participants will attend two booster sessions [13].

Experimental Group B: CMT/soft tissue techniques plus sham DMT Program

The experimental group B will receive the same cervical manual therapy techniques as the experimental group A, for 20 minutes plus sham diaphragmatic manual techniques with ultrasound for 10 minutes. Inactive ultrasound will be applied in the same patient position as for the experimental group A for 10 minutes, as a placebo treatment. The

chronometer of the ultrasound will operate and at the end of the time, a buzzer will sound [14]. Soft tissue techniques will be additionally applied for 10 minutes.

Active Control Group C: Conventional Physiotherapy (CP) Program

The Control Group C will receive a conventional physiotherapy program (CP). Initially, Transcutaneous Electrical Nerve Stimulation (TENS) will be applied with a pulse duration of 250 microseconds at a frequency of 80 Hz for 15 minutes in the suboccipital region and the trapezius bilaterally. Subsequently, microwave pulsed diathermy will be applied for 10 minutes. Soft tissue techniques (massage) will be additionally applied for 15 minutes. Massage therapy, included gliding and kneading techniques, will be applied over the trapezius (upper, lower, and middle fibers), splenius capitis, and levator scapulae muscles [15].

REFERENCES

[1] Hing W, Hall T, Mulligan B. The Mulligan concept of manual therapy. Textbook of techniques. 2nd edition. Elsevier, Australia, 2020.

[2] Buyukturan O, Buyukturan B, Sas S, Karartı C, Ceylan İ. The Effect of Mulligan Mobilization Technique in Older Adults with Neck Pain: A Randomized Controlled, Double-Blind Study. *Pain Res Manag.* 2018:2856375. https://doi.org/10.1155/2018/2856375.

[3] Exelby L. The Mulligan concept: its application in the management of spinal conditions. *Man Ther.* 2002 May;7(2):64-70. https://doi.org/10.1054/math.2001.0435.

[4] Wilson, E. The Mulligan concept: NAGS, SNAGS and mobilizations with movement. *J Bodyw Mov Ther.* 2001, 5, 81-89. https://doi.org/10.1054/jbmt.2000.0191.

[5] Chaitow L, Bradley D, Gilbert C, Bartley J, Peters D. Recognizing and treating breathing disorders: a multidisciplinary approach. 2nd ed. London: Elsevier Health Sciences UK; 2014.

[6] Lewit K. Manipulative Therapy Musculoskeletal Medicine: Churchill Livingstone Elsevier 2010 p.293-4, https://doi.org/10.1016/B978-0-7020-3056-7.X0001-0.

[7] Bennett, S., Siritaratiwat, W., Tanrangka, N., Bennett, M. J., & Kanpittaya, J. Effectiveness of the manual diaphragmatic stretching technique on respiratory function in cerebral palsy: A randomised controlled trial. *Respir Med.* 2021, 184, 106443. https://doi.org/10.1016/j.rmed.2021.106443

[8] Bruton A, Lee A, Yardley L, et al. Physiotherapy breathing retraining for asthma: a randomised controlled trial. *Lancet Respir Med*. 2018;6(1):19-28. https://doi.org/10.1016/S2213-2600(17)30474-5.

[9] Holloway EA, & West RJ. Integrated breathing and relaxation training (the Papworth method) for adults with asthma in primary care: a randomised controlled trial. Thorax. 2007, 62(12), 1039–1042. https://doi.org/10.1136/thx.2006.076430.

[10] Grammatopoulou, E. P., Skordilis, E. K., Stavrou, N., Myrianthefs, P., Karteroliotis, K., Baltopoulos, G., & Koutsouki, D. The effect of physiotherapy-based breathing retraining on asthma control. *The Journal of asthma : official journal of the Association for the Care of Asthma*, 2011, 48(6), 593–601. https://doi.org/10.3109/02770903.2011.587583

[11] Jafari, H., Gholamrezaei, A., Franssen, M., Van Oudenhove, L., Aziz, Q., Van den Bergh, O., Vlaeyen, J., & Van Diest, I. Can Slow Deep Breathing Reduce Pain? An Experimental Study Exploring Mechanisms. *J Pain*. 2020, 21(9-10), 1018–1030. https://doi.org/10.1016/j.jpain.2019.12.010

[12] Jordan, J. L., Holden, M. A., Mason, E. E., & Foster, N. E. Interventions to improve adherence to exercise for chronic musculoskeletal pain in adults. *The Cochrane database of systematic reviews*, 2010(1), CD005956. https://doi.org/10.1002/14651858.CD005956.pub2

[13] Cinthuja, P., Krishnamoorthy, N. & Shivapatham, G. Effective interventions to improve long-term physiotherapy exercise adherence among patients with lower limb osteoarthritis. A systematic review. *BMC Musculoskelet Disord.* 2022, 23, 147 https://doi.org/10.1186/s12891-022-05050-0

[14] Macías E, Torres Sánchez, I., Cabrera-Martos, I., Ortiz, A., López, L., & Valenza, M. Effects of manual therapy on the diaphragm in asthmatic patients: A randomized pilot study. *Int J Osteopath Med.* 2018, 29, 26-31. https://doi.org/10.1016/j.ijosm.2018.07.006.

[15] Rodríguez-Huguet M, Rodríguez-Almagro D, Rodríguez-Huguet P, Martín-Valero R, Lomas-Vega R. Treatment of Neck Pain With Myofascial Therapies: A Single Blind Randomized Controlled Trial. *J Manipulative Physiol Ther*. 2020, 43, 2, 160-170. https://doi.org/10.1016/j.jmpt.2019.12.001.
