# Supplementary material for: The Effectiveness of Manual Therapy in the Cervical Spine and Diaphragm, in Combination with Breathing Reeducation Exercises, in Patients with Non-Specific Chronic Neck Pain: Protocol for Development of Outcome Measures and a Randomized Controlled Trial
Source: Diagnostics (Basel). 2022 Nov 4;12(11):2690. doi: 10.3390/diagnostics12112690 (PMC9689657; doi:10.3390/diagnostics12112690)
Supplement: Supplementary file 1 [file diagnostics-12-02690-s001.zip › diagnostics-1936124-Table S1.pdf]

Table S1. Schedule of enrollment, intervention and assessments. BREx: Breathing Reeducation Exercises, CP: Conventional Physiotherapy, CMT: Cervical Manual Therapy, DMT: Diaphragmatic Manual Therapy, sDMT: Sham Diaphragmatic Manual Therapy, STT: Soft Tissue Techniques

	**STUDY PERIOD**				
	**Enrollment**	**Allocation**	**Post-allocation**		**Close-out**
**TIMEPOINT**	***$-t_1$***	**Baseline (0)**	***0-4 weeks***	***4-12 weeks***	***12 week endpoint***
**ENROLLMENT:**					
**Eligibility screen**	X				
**Informed consent**	X				
Randomization	X				
**Allocation**		X			
**INTERVENTIONS:**					
Intervention A: CMT-DMT- STT- BREx			↔		
Intervention B: CMT- sDMT- STT			↔		
Intervention C: CP			↔		
All groups: Self-managed exercises				↔	
**ASSESSMENTS:**					
Baseline		X			
4 weeks			X		
12 weeks					X
